# High-precision speckle-tracking X-ray imaging with adaptive subset size choices

**DOI:** 10.1038/s41598-020-71158-9

**Published:** 2020-08-28

**Authors:** Naxi Tian, Hui Jiang, Aiguo Li, Dongxu Liang, Fucheng Yu

**Affiliations:** 1 Shanghai Institute of Applied Physics, Chinese Academy of Sciences, Jialuo Road 2019, Jiading District, Shanghai, 201800 China; 2 Shanghai Synchrotron Radiation Facility, Shanghai Advanced Research Institute, Chinese Academy of Sciences, Zhangheng Road 239, Pudong District, Shanghai, 201204 China; 3 University of Chinese Academy of Sciences, Beijing, 100049 China

**Keywords:** X-rays, Imaging and sensing

## Abstract

Speckle-tracking imaging has the advantages of simple setup and high-sensitivity to slowly varying phase gradients. Subset size choice is regarded as a trade-off problem for speckle-tracking X-ray imaging where one needs to balance the spatial resolution and accuracy, where the subset was defined as the region of interest of windowing choice for digital image correlation algorithm. An adaptive subset size choice method based on a Fourier transform for effectively detecting sample phase information without foreknowledge of the sample structure is presented in this study. The speckle-tracking phase-contrast and the form of dark-field imaging based on this method have the advantages of (i) high resolution and time saving compared to large subset choice and (ii) partially improvement the influence from experimental noises, background fluctuations, and false signals compared to small subset choice at the same time. This method has proven to be particularly robust in the experimental condition of poor signal-to-noise ratio. The proposed method may be expanded to all speckle-based imaging methods and other imaging techniques based on the subset or window matching.

## Introduction

The speckle-based X-ray imaging technique^1–3^ has been developed recently due to its many advantages compared to conventional propagation-based^4^ or grating-based^5^ imaging techniques. Speckle-based X-ray imaging uses a random phase object such as sandpaper or a biological membrane to modulate the X-ray wavefront. A pixel detector is then used to record the speckle patterns with and without a sample. By analysing these two patterns, the information from sample-induced distortion can be obtained. This technique can thus reconstruct two-dimensional absorption, phase-gradient and dark-field information in a double-exposure measurement^6–9^. Compared to propagation-based imaging, speckle-based imaging has proven to yield significant improvement in detecting slowly varying phase gradients, since it reconstructs first-order derivative phases. Compared to grating-based imaging, speckle-based imaging has simpler and cheaper experimental setup, and it avoids some problems such as phase wrapping and low transmission^10^. Speckle-based X-ray imaging can also be used in polychromatic and partial coherent situations directly^11^, conditions that produce significant limits and reconstruction errors for ptychography imaging^12^.

While powerful, speckle-based imaging has its inherent downsides or pitfalls, similar to every imaging modality. The first common downside is that speckle-based imaging is insensitive to high-frequency features, but this can be partially improved by removing the effect of free-space propagation from the speckle pattern in order to produce an edge enhancement^13^. The second main pitfall in speckle-based imaging is the choice of the subset size in the necessary digital image correlation (DIC) algorithm^14^. Here the subset was defined as the region of interest or windowing choice for DIC. A larger subset size can accurately distinguish itself from other subsets but a smaller subset size affords higher spatial resolution. These two conflicting demands force researchers to manually attempt to choose a relatively satisfying subset size for a specific sample. Pan et al. used the sum of the square of the subset intensity gradient (SSSIG) as a criterion to select the subset size^15^, but for a complicated sample this study did not design any thresholds for the criterion to suit different positions. Zhou et al. used a contrast-to-noise ratio (CNR) to simulate the influence from the spatial frequency^16^. This parameter was sensitive to the subset size. However, giving a criterion to choose the subset size based on an actual sample was not attempted in this work.

One idea for solving this trade-off problem of subset size choice is to design an adaptive subset to suit different positions in a speckle pattern. This idea was first proposed in signal or image processing. An adaptive Fourier transform was used to optimize the bias-to-variance trade-off for time-varying signals with a minimal mean squared error^17^. In three-dimensional surface shape measurement, the windowed Fourier transform and wavelet transform methods were used to extract phase information from a single-fringe pattern^18^. An iterative adaptive cross-correlation was used to improve the accuracy of the DIC for wavefront sensing with high subpixel accuracy^19,20^. This technique focused on the improvement of the shift estimate accuracy by fitting the peaks of the cross-correlation or the phase slopes with the variation of the subset size, but it did not build a relationship between the subset size and the sample and it had a large associated computation time.

In this study, we present a novel method based on Fourier analysis for speckle-tracking X-ray imaging. The use of an adaptive subset size choice (ASC) proves to be effective in improving the resolution, decreasing background fluctuations and false signals, and reducing the required computation time.

## Methods and simulations

In the X-ray speckle-tracking imaging mode, the superposition of the interference of the weak scattered beams from a diffuser and stronger transmitted beams produced a speckle intensity distribution on the detection plane. The sandpaper can be described as consisting of many grains. Based on the intensity extractions *f*_*o*_ and *f*_*s*_ obtained from two speckle patterns, with and without a sample, and using the two-dimensional DIC method^10^, the shifts of the maximum cross-correlation coefficient (MCCC) *ε* = argmax(*f*_0_ $\otimes$* f*_s_) in both directions can be obtained. These shifts are proportional to the respective phase gradients. The symbol $\otimes$ marks all correlation operations. The *f*_*o*_ and *f*_*s*_ have different expressions for different cross-correlation calculations. The MCCC was shown to have a relationship to the dark-field signal with different expression forms^7,21^, which is reduced by small-angle scattering from the sample structure. The cross-correlation criterion has many representations, of which the zero-normalized cross-correlation (ZNCC) or the zero-normalized sum of the squared differences (ZNSSD) are considered the most robust and reliable approaches as a two-dimensional digital image correlation method^14^, and they are widely used at present. For this criterion, *f*_*o*_ and *f*_*s*_ can be expressed as follows:

1 $$ f_{{0/{\text{s}}}} (x,z) = \left( {\frac{{I_{{0/{\text{s}}}} (x,z) - \overline{{I_{{0/{\text{s}}}} }} }}{{\Delta I_{{0/{\text{s}}}} }}} \right)w(x,z) = g_{{0/{\text{s}}}} (x,z)w(x,z), $$

where *I*_0_ and *I*_s_ are the intensity patterns without and with a sample, the overline and the symbol Δ indicate the mean value and the sum of squares of the deviation from the mean of the intensity patterns, respectively, and *w* is the subset window function. Here, the subset window function is the rectangle function *w*(*x*,*z*) = rect(*x*/*M*, *z*/*N*), which is unity for |*x*| ≤ 1/2 and |*z*| ≤ 1/2 but zero otherwise, where *M* × *N* defines the subset size. In previous studies, equal values of *M* and *N* were chosen for the side lengths of the subsets.

**Figure 1:**
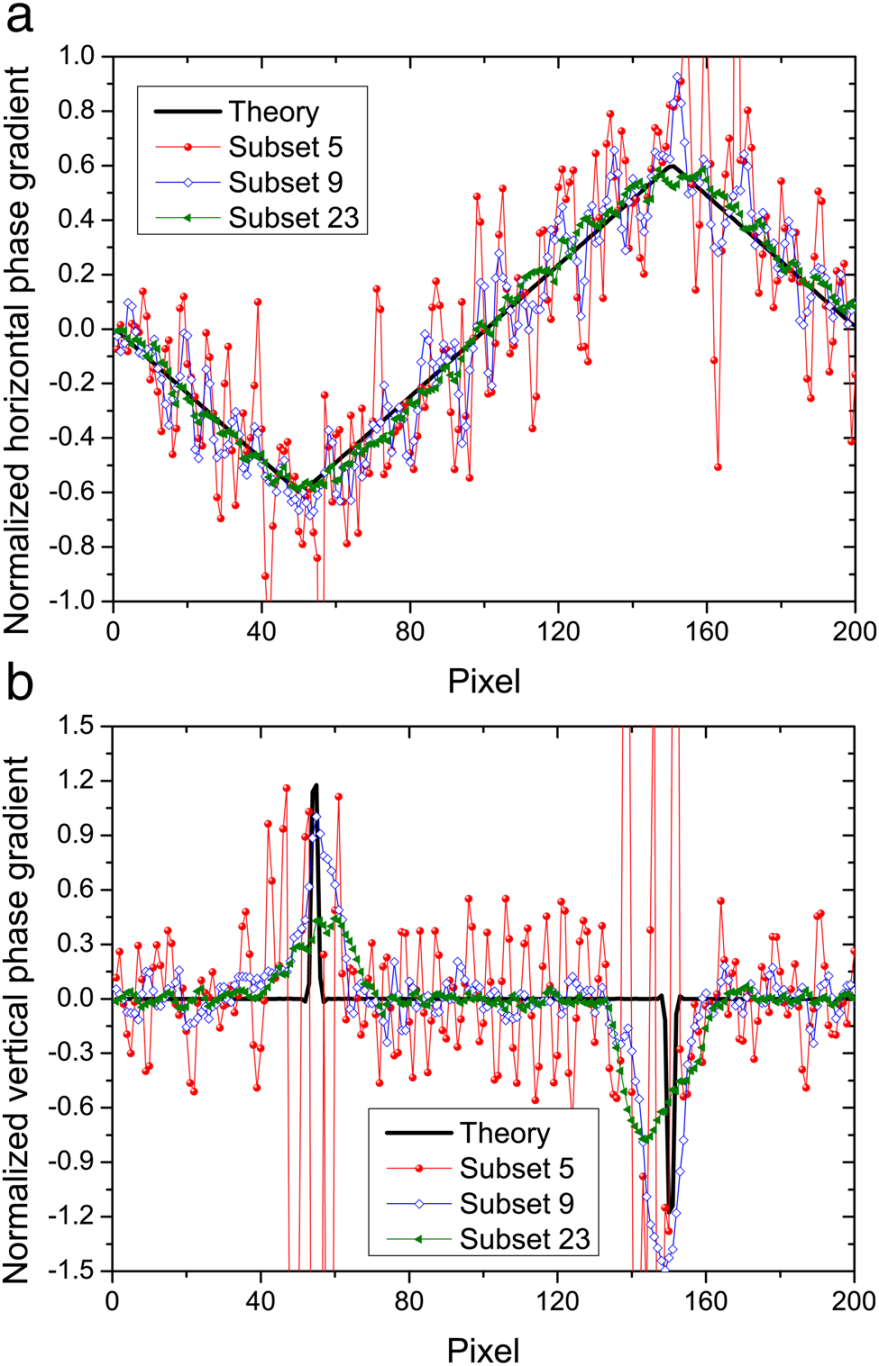
Comparison of the (**a**) horizontal and (**b**) vertical phase gradients with different subset size choices calculated from the simulated speckle patterns with and without a sample.

In order to study the criterion for the subset choice, we designed a simulator to producing speckle patterns. The radii of the grain sizes and the positions of the grains satisfied two normal distributions. In this simulation, the mean radius of the grain size of the sandpaper was 0.25 μm and the standard deviations of the radii and the positions in two dimensions were 30% and 50% of the mean grain size of the sandpaper, respectively, based on the estimation of real sandpaper. The horizontal phase gradient of the sample was designed to be triangular, as is shown in Fig. 1a. The propagation process was simulated by a Fresnel approximation of the diffraction integral^22^ at an energy of 17 keV. The speckle patterns with 220 × 220 pixels with and without a sample were recorded 1.6 m downstream of the sample with a pixel size of 0.1 μm.

In our simulation, the side length values of the subset, *M* and *N* pixels, were kept equal. Figure 1 demonstrates the phase gradients along both directions which were calculated with different subset size choices. When the subset size is smaller than five pixels, that is, the average size of a grain, all results have serious oscillations. Our previous study^23^ presents a more detailed explanation of the relationship between grain size of sandpaper and subsets. As the subset size increases, the oscillation of the horizontal phase gradient becomes smaller and smaller, approaching the real phase gradient. The root-mean-square (RMS) error reduces and approaches a constant of 11% when the subset size is larger than 23 pixels. The vertical phase gradient includes high-frequency step signals. When the subset size is around 7–9 pixels, the edges of the sample can be distinguished. With an increase of the subset size, the edge signals broaden and shift into the sample zone. In this case, the resolution decreases and it is hard to distinguish the correct sample edges. Compared to the horizontal phase gradient with its relatively low spatial frequency, the effective subset size choice for the high-frequency vertical phase gradient is only in a very narrow range.

**Figure 2:**
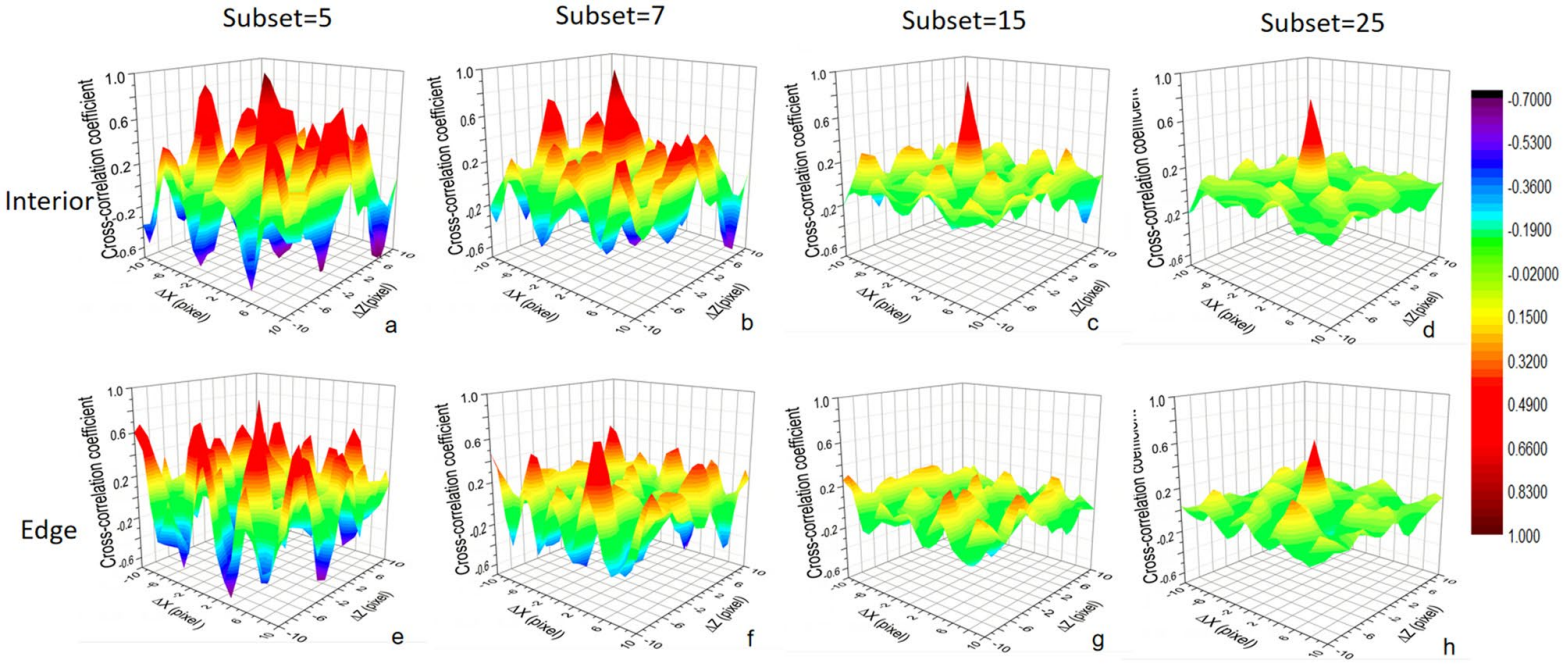
Cross-correlation maps with different subset choices from 5 to 25 inside the sample (upper) and at the edge of the sample (lower).

By considering the process of the DIC algorithm, we clearly understand the reason why the phenomenon in the simulation occurs. For any window centre (*x*′, *z*′), in order to accurately search out the maximum value *c*_max_ in a cross-correlation map *c*(*x*, *z*, *x*′, *z*′) = *f*_0_ $\otimes$*f*_s_, the ideal case of the function *c* has a sharp main peak (Dirac function in theory), which is similar to the Fig. 2d. The locations and the number of satellite peaks in the cross-correlation map depend on the distribution of the sandpaper grains since the grains exist as markers in the speckle tracking imaging technique. If there are two or more peaks with similar intensities in the cross-correlation map due to the problem of parameter selection and the sample itself, the algorithm may fail and incorrect shift information may be found, as is shown in Fig. 2a,e. The result is often characterized as a false or discontinuous signal in the imaging. This phenomenon is very common in high-frequency signal detection while using an unsuitable subset size, because high-frequency regions and low-frequency regions relate to very different phase shifts in a subset region and they display significant competition for searching out a unique maximum *ε* using the DIC algorithm. An excessive subset dilutes the weight of the high-frequency components in the algorithm and produces an over-wide weak single peak (Fig. 2g) or even finds an incorrect main peak (Fig. 2h). In Fig. 2h, this incorrect main peak is at (0, 0) which means that algorithm judges that this place is out of the sample. This is the reason that the edge shifts into the sample using an excessive subset as is shown in Fig. 1b. A subset that is too small causes itself to not have enough features as a marker so that it is difficult to distinguish and it is easily influenced by optical blurs and noises, as is shown in Fig. 2a, e. The demonstration of searching process for MCCC in the cross-correlation map with different subset choices inside and at the edge of the sample is shown in the Fig. 2. In summary, for the interior of sample, large subset choice produces single sharp peak so that searches out the right shift, but at the edge of sample, too small (Fig. 2e) and too large (Fig. 2h) subset choices both cannot search out the right peak position (Fig. 2f).

It is clear that the subset choice is related to the local spatial frequency of sample. Thus, we need to build a relationship between the cross-correlation coefficient and the spatial frequency of the sample. Based on the Fourier transform properties for correlation theorems, a two-dimensional Fourier transform of the cross-correlation coefficient of two patterns can be expressed as follows:

2 $$ C(u,v,x^{\prime } ,z^{\prime } ) = \Im \left[ {f_{0} \otimes f_{s} } \right](u,v,x^{\prime } ,z^{\prime } ) = F_{0} *(u,v,x^{\prime } ,z^{\prime } )F_{s} (u,v,x^{\prime } ,z^{\prime } ), $$

where * is the conjugate operator, and *C* and *F* are the Fourier transforms of *c* and *f*. If we assume a shift of the MCCC *ε* = (*x*_0_*,z*_0_), according to the Fourier shift property:

3 $$ F_{s} (u,v,x^{\prime } ,z^{\prime } ) = F_{0} (u,v,x^{\prime } ,z^{\prime } ){\text{e}}^{{ - i2{\uppi }(ux_{0} + vz_{0} )}} . $$

Thus, the shift of the MCCC *ε* can be directly related to the phase angle:

4 $$ \phi (u,v) = - 2{\uppi }(ux_{0} + vz_{0} ) = \arctan [{\text{Re}} (C)/{\text{Im}} (C)]. $$

**Figure 3:**
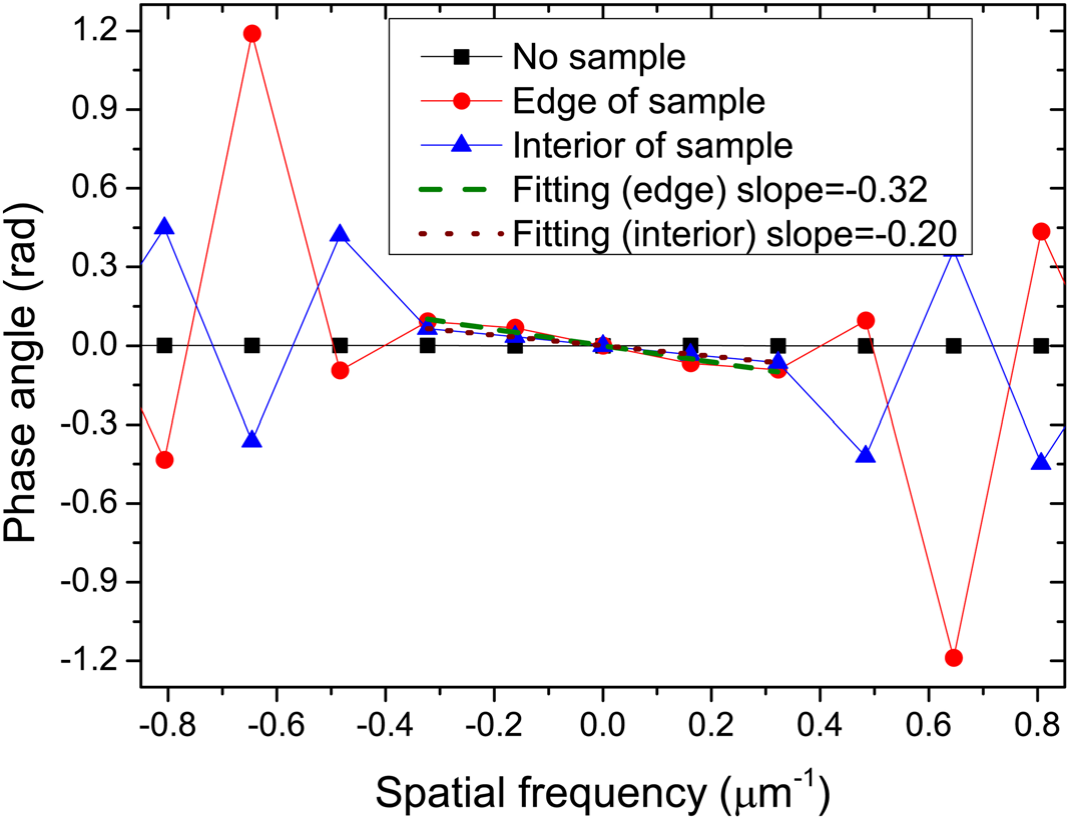
Phase angles along the vertical direction calculated at the different positions (out of, edge of and interior of sample) and their fitting curves at low spatial frequency region from − 0.4 to 0.4 μm.

For a set of unique shifts (*x*_0_*,z*_0_), the phase angle is a plane function. However, this ideal situation only happens in the translation of the same pattern. When introducing a sample, the effects on the speckle pattern vary dramatically at different frequencies. Normally the DIC algorithm only judges the macroscopic level, namely, the lowest-frequency shifts in the subset region. Based on the abovementioned discussion, a successful algorithm corresponds to a set of significantly optimal shifts. It is reflected in the phase angle is that there is remarkable monotonicity and even linearity in the low-frequency region. Figure 3 demonstrates the phase angles along the vertical direction at the different positions relative to the sample. The slopes of fitting curves the lowest-frequency region from − 0.4 to 0.4 μm^−1^, correspond to the shift of the MCCC. If there are two peaks or several peaks having similar intensities in the correlation map, the lowest spatial frequency region of the phase angle shows a jagged and disorderly shape. The shifts *x*_0_ and *z*_0_ are directly proportional to the slopes in the corresponding directions.

**Figure 4:**
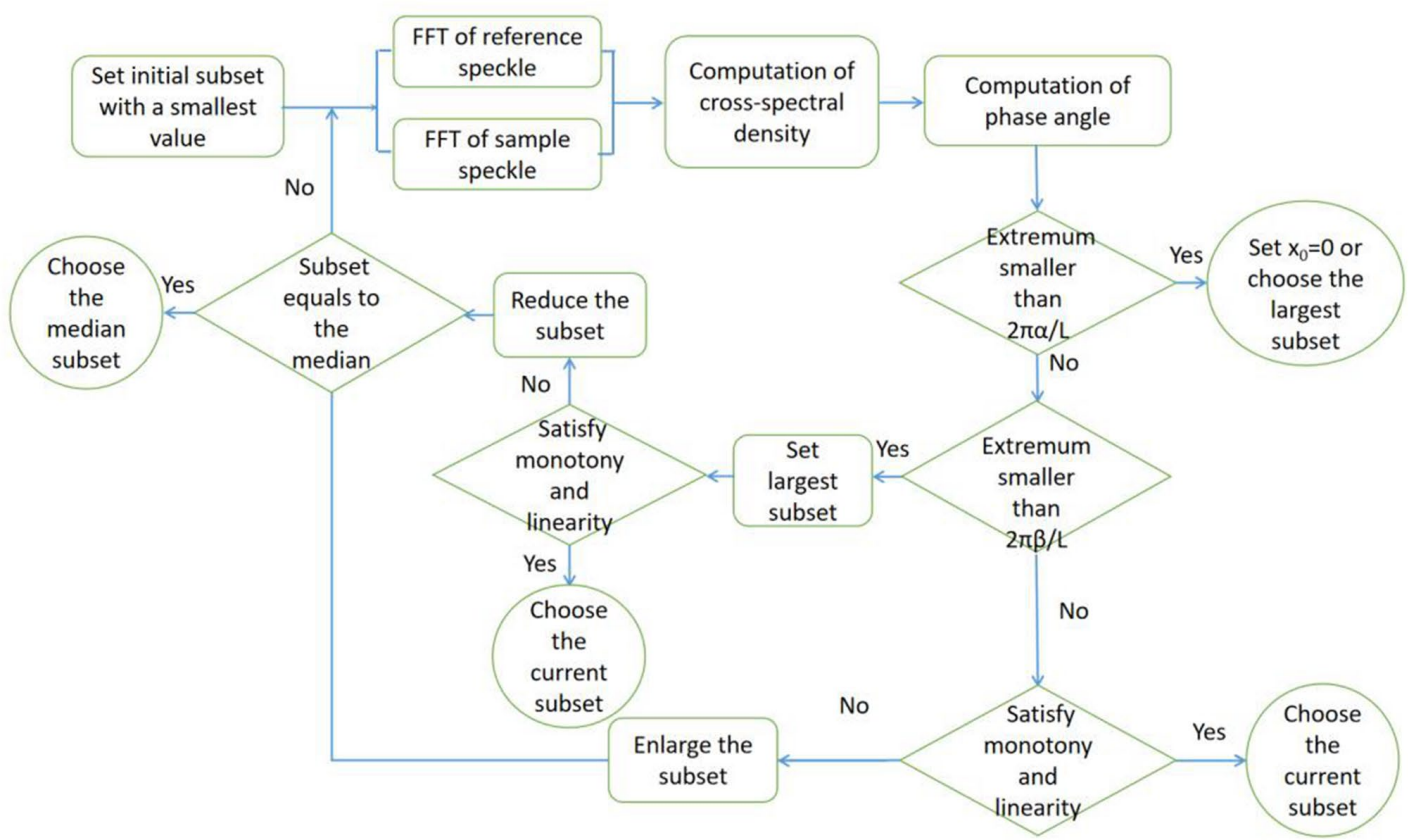
Flow chart of the algorithm for searching out an optimal subset size.

For a region without a sample or without a phase change, since the slope corresponding to the shift is small enough and random, the values of the phase angle in the entire medium and the low-frequency region are very small, as is shown the curve of “no sample” in Fig. 3. Assuming that the sampling of the spatial frequency in any direction is *L*, criterion 1 $\phi_{1} < 2{\uppi }\alpha /L$ can be used to judge this kind of region. In this formula, *α* is a phase gradient sensitivity factor, typically 0.5–3, depending on the signal-to-noise ratio (SNR) and accuracy of the sub-pixel algorithm^14^ like 0.01–0.1 pixel. High algorithm accuracy and high SNR ensure a small *α*. In general, a relatively small value can avoid the loss of sample signal. When ${\phi }_{1}$ is smaller than the value*α*, the side length of subset can be set to the given largest value which includes at least 4–5 grains to pursuit high accuracy or the shifts in this area can even be set to zero directly and the DIC algorithm can be skipped to save calculation time. As the shift increases, the phase angle becomes steeper. In a given low-frequency region, by judging whether the phase angle has stable monotony and satisfies linearity along one direction, we can judge whether the selected subset size is reasonable. A reasonable subset ensures that the central signal within the subset can dominate the optimization of the cross-correlation coefficient, whether it is a slowly varying signal or a high-frequency signal. Normally, this reasonable subset size has a range of values dependent on the spatial frequency. Based on the simulation, for a high-frequency signal, the reasonable subset is smaller, whereas the relatively large subsets have to be selected to reduce the RMS error to the theoretical phase gradient for a slowly varying signal. A given value of the fitted slope or the local extreme $\phi_{2} < 2{\uppi }\beta /L$ (criterion 2) in the low-frequency region can be used to distinguish between a high-frequency signal and a slowly varying signal, where *β* is a threshold factor corresponding to the shift of 0.5–1 pixel, empirically 5–15, depending on the SNR. Lower SNR corresponds to a greater *β* value. Although the factors *α* and *β* need to be tuned, the setting of these two factors is not very sensitive, but slightly affects the accuracy of the results. As can be seen in Fig. 3, the threshold can distinguish between two different slopes of − 0.32 and − 0.20, corresponding to the edge and interior of the sample. For the high-frequency signal, the initial subset is the given smallest subset, and then the subset size increases until the phase angles satisfies the monotony and linearity conditions. Based on sampling theorem, the smallest size should have been larger than five pixels. However, the optimal subset size for slowly varying signals is derived from a given largest subset when the criterion 2 is triggered. Phase unwrapping is used if the wrapped phase angle is found in the low-frequency region. In order to satisfy the continuity of the two spatial frequency bands, the median of the subset size range is set as the finishing point of the optimizations, where the median means the average of the given smallest and largest subset sizes. Since the finishing points of the criterions of these two spatial frequency bands are same, the deviation of the value *β* has little effect on the reconstruction result based on a large number of simulations. Figure 4 presents the basic procedure for searching out an optimal subset size. The entire ASC procedure can automatically implement before the DIC algorithm based on the given threshold conditions by estimating the SNR in an experiment.

**Figure 5:**
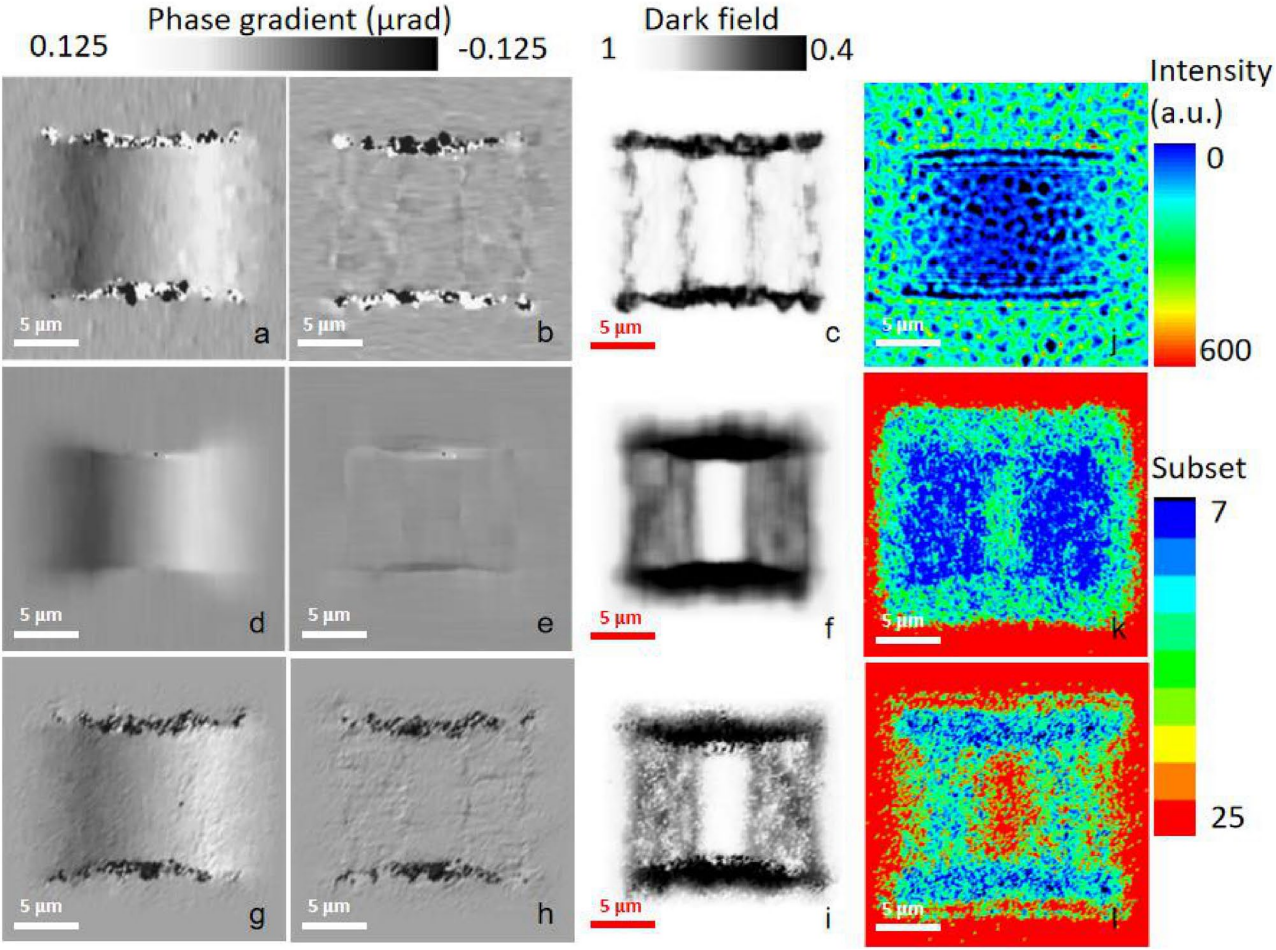
Simulated sample: the (**a**) horizontal and (**b**) vertical phase gradients and (**c**) the dark field for the subset size of 7 × 7; the (**d**) horizontal and (**e**) vertical phase gradients and (**f**) dark field for the subset size of 25 × 25; the (**g**) horizontal and (**h**) vertical phase gradients and (**i**) dark field for the ASC; (**j**) speckle patterns with a sample; the (**k**) horizontal and (**l**) vertical side length of the subset.

**Figure 6:**
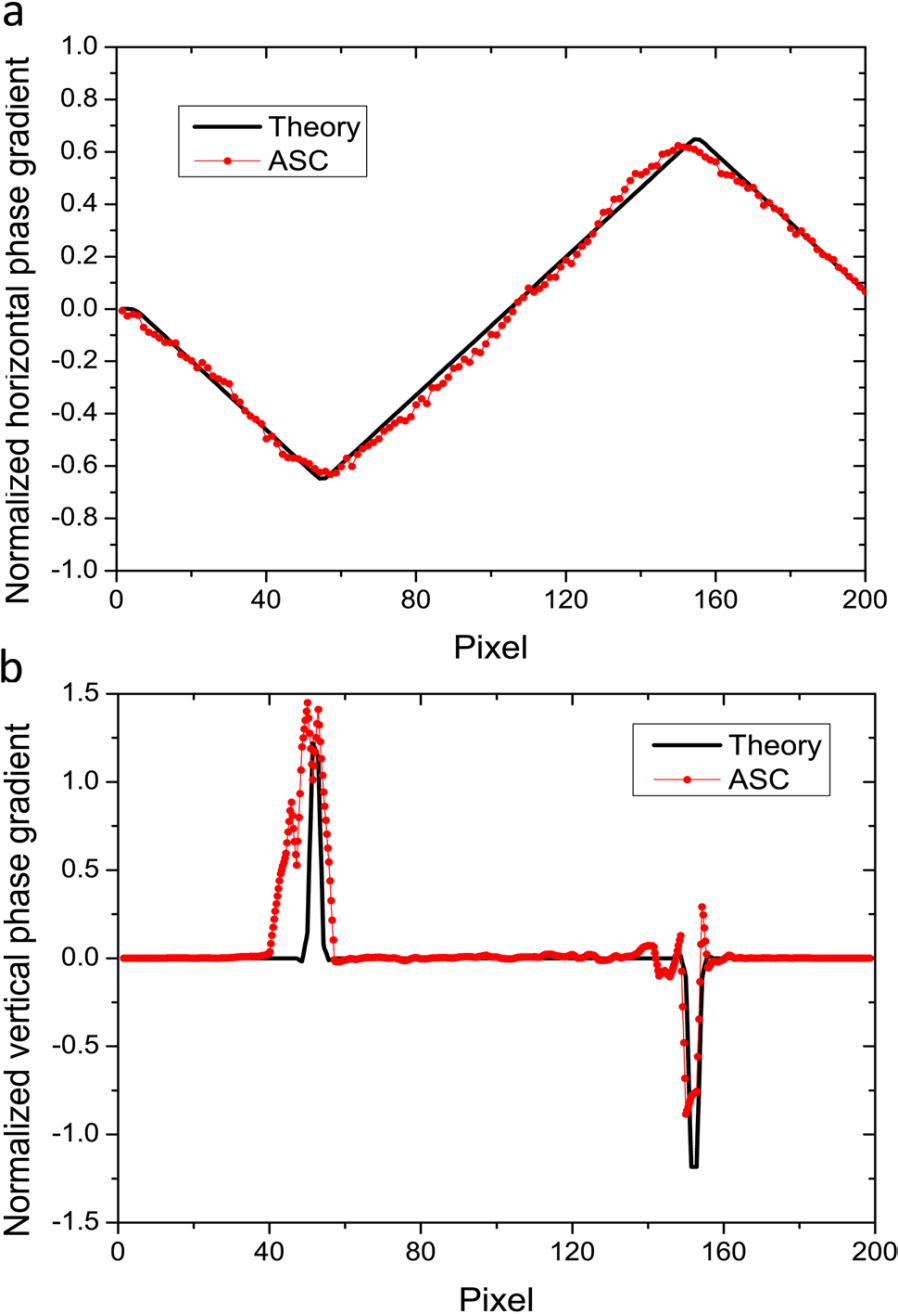
Comparison of the (**a**) horizontal and (**b**) vertical phase gradients with ASC method and the theoretical curves calculated from the simulated speckle patterns with and without a sample.

Figure 5 shows the comparison of the criteria of the fixed subset sizes and the variable subset sizes. Figure 5j presents the simulated speckle pattern with a sample. Figure 5k,l show the subset sizes based on ASC in both directions, which clearly outline the phase changes of the simulated sample. The subset size is obviously related to the spatial frequency of the phase gradients. The phase-contrast images based on our subset choice shown in Fig. 5g,h improved the accuracy and smoothness of the slowly varying signals compared to the results with the smallest subsets (Fig. 5a, b). They also completely overcame the situation of the shift of the high-frequency edge signals while using a large subset, as shown in the vertical direction in Fig. 5d,e. Figure 6 indicate the phase gradients calculated using ASC method and their theoretical curves along both directions. It is clear that the ASC method makes that the slow varying and high-frequency signals get good reconstructions at the same time. Table 1 compares the RMS errors for horizontal slowly varying signals and the edge deviations for vertical high-frequency signals based on different subset size choices. Compared to the SSSIG criterion, our criterion based on a Fourier transform has better sensitivity for distinguishing between a sample and sandpaper grain, and it saves significant computation time. The ASC based entirely on actual sample information causes the subset choice and the algorithm to no longer have periodicity, which also brings other corresponding advantages and disadvantages. One advantage of this is that the false fringes from the algorithm disappear in Fig. 5g,h. The disadvantage of this is that the continuity of the dark-field image, as is shown in Fig. 5i, becomes partly weakened because the variable subset size choice produces additional calculation fluctuations for the high-sensitivity correlation coefficients.

**Table 1:** Comparison of the reconstruction errors in both orthogonal directions and the computation time with different methods of subset size choice.

Subset size choice	RMS error for horizontal signals (%)	Edge deviation for vertical signals (pixel)	Normalized computation time
7 × 7	33	< 0.5	1
9 × 9	23	< 1	1.13
25 × 25	11	6.5	1.57
Adaptive based on SSSIG	13	1.5	1.97
Adaptive based on phase angle slope	13	< 0.5	1.32

**Figure 7:**
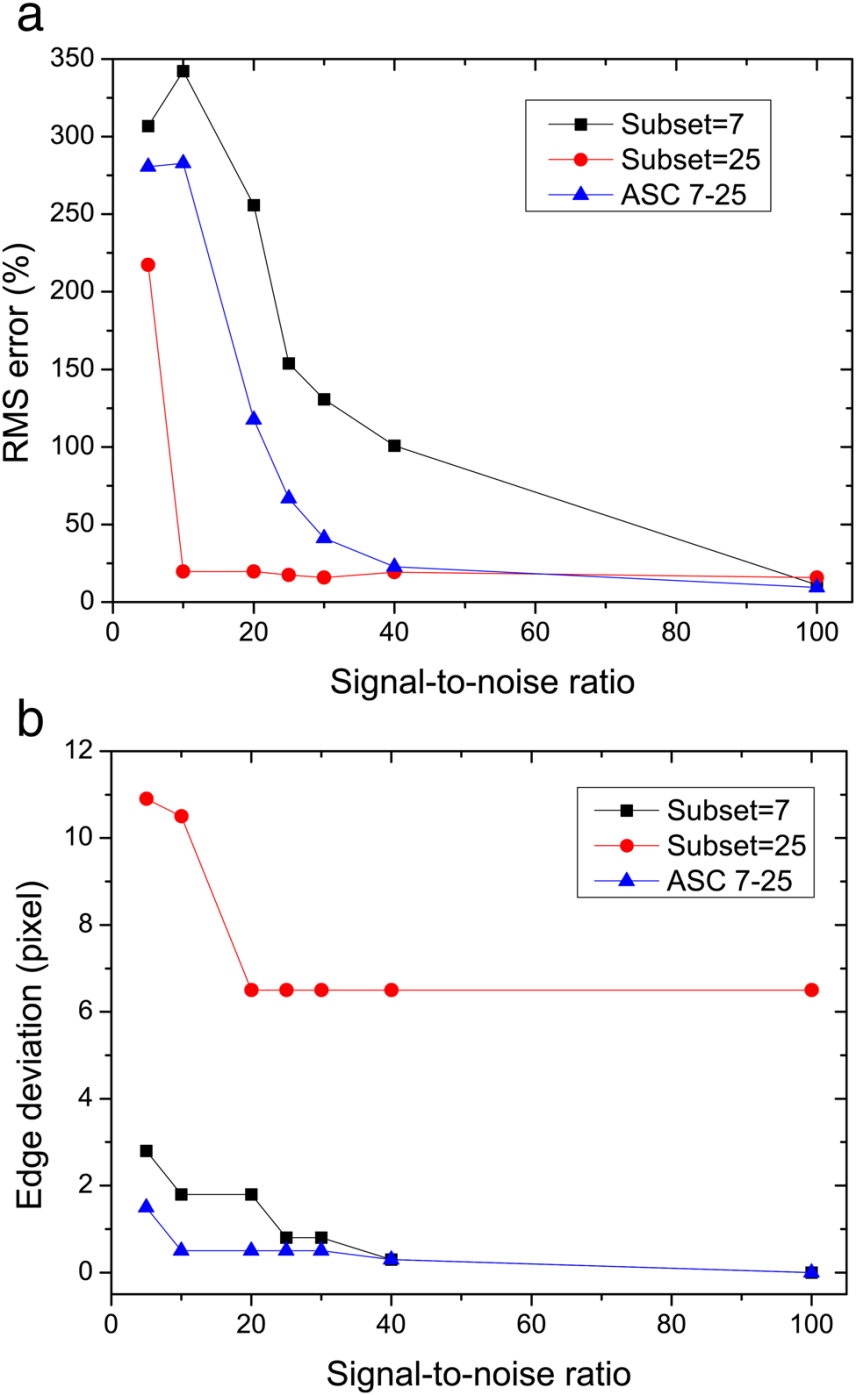
RMS error for horizontal signal and the edge deviation for vertical signal versus the SNR.

We also simulated the influences of the Gaussian noises with the signal-to-noise ratio (SNR) from 5 to 100. As is shown in Fig. 7a, the algorithm using small subset suffers serious disturbance from noises and reconstruction RMS error keeps large even if the SNR reaches 40. As a contrast, the algorithm using the large subset almost is immune to the noises as long as the SNR is greater than 10. The high level of robustness with respect to noise for the algorithm using ASC is in between and can be accepted when the SNR is greater than 25, and decreases to the level of large subset when the SNR is greater than 40. For high-frequency edge signal, the influences of noises are similar using different subset choices. As is shown in Fig. 7b, as long as the SNR is greater than 20, the edge deviation is in agreement with the results without noises in Table 1. As we all know, the SNR is an important factor that affects the imaging quality. The robustness with respect to noise using ASC is a significant advantage over using a fixed small subset in the condition of relatively poor SNR.

## Experiments

We also measured some actual samples to prove our techniques. The experiments using polymethyl methacrylate (PMMA) spheres with a diameter of 1.05 mm and a JIMA tungsten resolution target (RT) (line grating structures in both orthogonal directions) with the thickness of 1.0 μm were performed at the BL13W imaging beamline and the BL09B measurement beamline, respectively. The sample, SiC sandpaper, and detector were arranged in an unfocused X-ray beam path. The detection system was a microscope objective lens system (Optique Peter) with a given magnification coupled to a CMOS camera (Hamamatsu). The main parameter details can be found in Table 2. Based on the SNRs in the experiments, the sensitive factor *α* and the threshold factor *β* were 0.5 and 5 for the PMMA and 2.5 and 8 for the resolution target.

**Table 2:** Experimental parameters of the speckle-tracking imaging measurements.

Sample	Sample-to-sandpaper distance (mm)	Energy (KeV)	Sample-to-detector distance (mm)	Sandpaper grain size (μm)	Effective pixel size (μm)	Exposure time (s)	SNR
PMMA	100	20	650	20	3.25	0.5	44
RT	312	10	1,258	1	0.65	10	21

## Results

**Figure 8:**
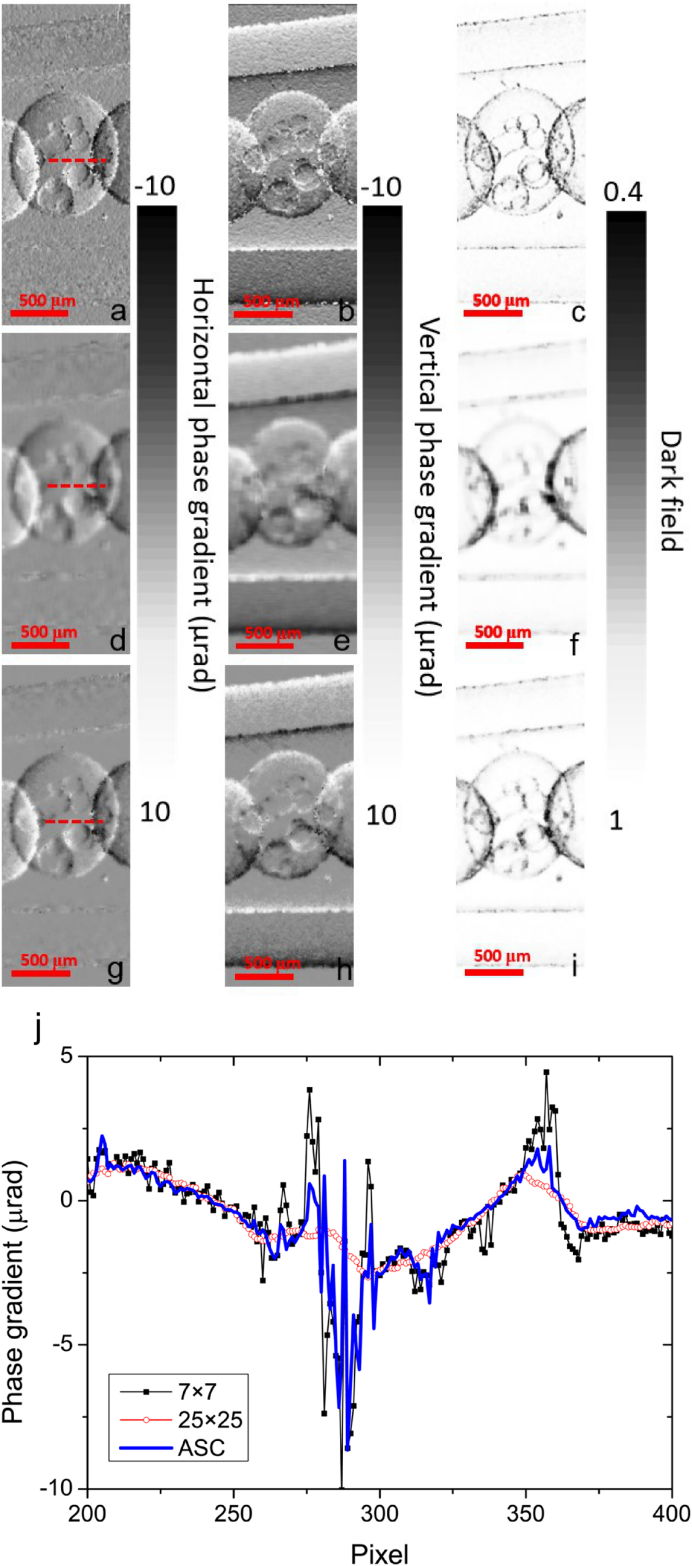
PMMA spheres were recorded by a CMOS camera with the detection field of 400 × 1,400 pixels. The (**a**) horizontal and (**b**) vertical phase gradients and (**c**) the dark field for the subset size of 7 × 7; the (**d**) horizontal and (**e**) vertical phase gradients and (**f**) the dark field for the subset size of 25 × 25; the (**g**) horizontal and (**h**) vertical phase gradients and (**i**) the dark field for the ASC; (**j**) comparison of the horizontal gradient profiles (red dashed lines) with different subset size choices in (**a**), (**d**), and (**g**).

Figure 8 show speckle-tracking phase-contrast and dark-field images of the PMMA spheres based on different subset size choices. This sample has obviously slow varying structures which is similar to the simulated sample. Using a small subset size has a strong edge-enhancement effect, as shown in Fig. 8a–c, but some fluctuations are introduced by the algorithm in the whole images. Due to high SNR of 44 and large phase gradient of sample, these background fluctuations can be accepted. The large subset size of 25 × 25 seriously deteriorates the high-frequency information at the sphere edges. Bubbles in the middle PMMA sphere are even harder to identify clearly. The ASC method almost retains most of the fine structural contrast and smooths slowly varying features and background noises. Figure 8j compares the horizontal phase gradient profiles marked as the red dashed lines in images. This measurement reveals that in the condition of high SNR, ASC method has the similar performance with the small subset choice.

**Figure 9:**
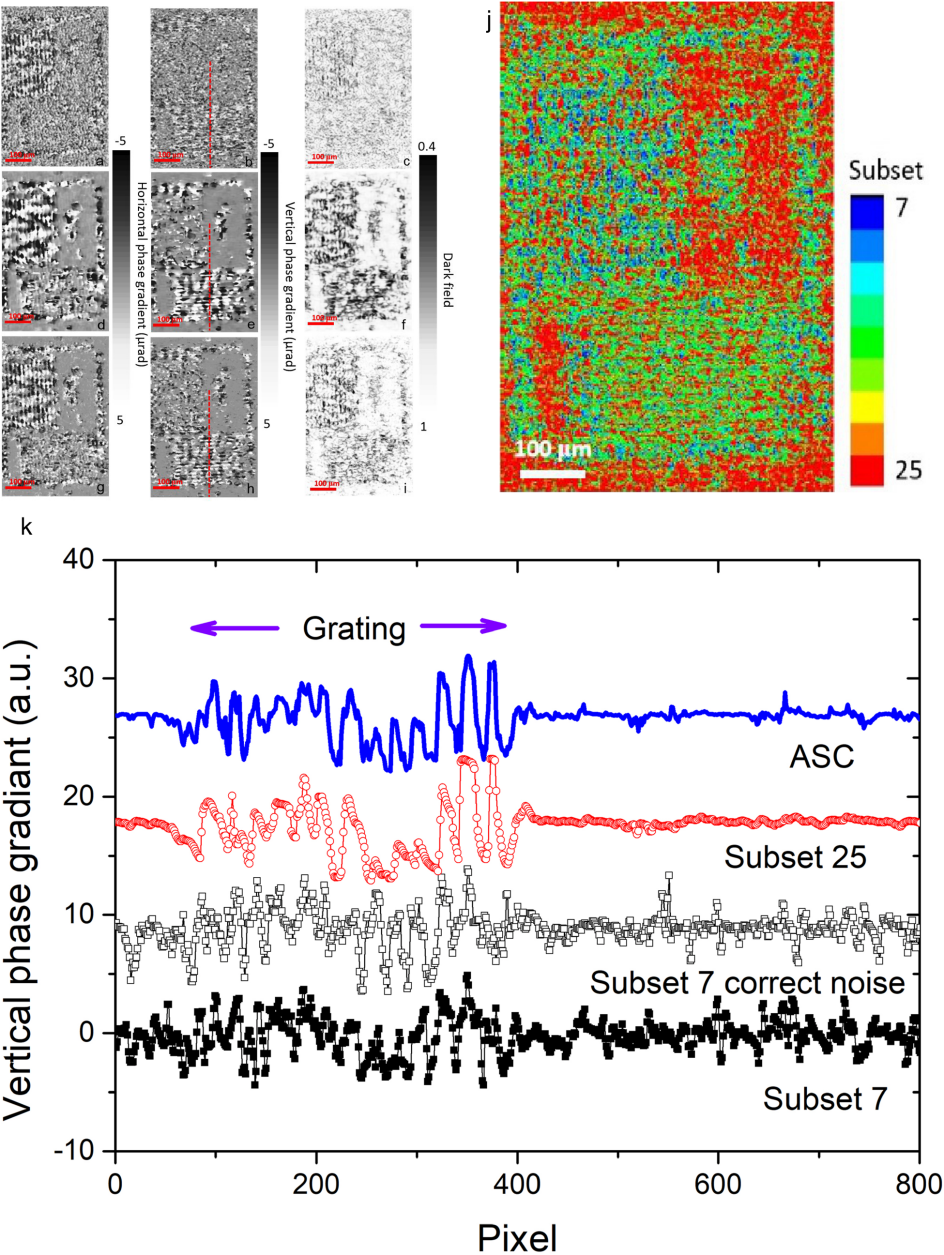
The resolution target was recorded by a CMOS camera with a resolution of 620 × 1,100 pixels. The (**a**) horizontal and (**b**) vertical phase gradients and (**c**) the dark field for the subset size of 7 × 7; the (**d**) horizontal and (**e**) vertical phase gradients and (**f**) the dark field for the subset size of 25 × 25; the (**g**) horizontal and (**h**) vertical phase gradients and (**i**) the dark field for the ASC; (**j**) vertical side length of the subset; (**k**) comparison of the vertical gradient profiles (red dashed lines) with different subset size choices in (**b**), (**e**) and (**h**) and the subset of 7 with denoising technique.

The measurement of RT performing at the bending magnet beamline is different. The weak flux with a long time exposure introduced more background noises with the poor SNR of 21. Based on the simulations in Fig. 7, this level of SNR may seriously affect the result of small subsets. Figure 8 show speckle-tracking images of the resolution target. The experiment verifies the simulated results. Compared to PMMA spheres, the resolution target has a much finer line grating structure of ~ 15 μm. Using a small subset size, such as 7 × 7 or 9 × 9, the line structures cannot be clearly distinguished from background fluctuations, as are shown in Fig. 9a–c. As can be seen in Fig. 9k, at a range of pixels of 400–800, the fluctuations are random but the sharp phase gradient peaks obviously confuse the real line grating structures of the resolution target at the range of pixels of 100–400. Some false peaks at pixels 160 and 220 also can be found. These fluctuations and false peaks can be eliminated by using large subset size. We also tried to use a denoising method reported in reference^24^ to correct the detector noise and beam fluctuations. Although the result (hollow square curve) is slightly improved, the fluctuations cannot be eliminated significantly. However, a large subset with the side length of 16.25 μm spans two adjacent line structures, which causes the phase gradient signal to broaden (Fig. 9d–f) and reduces the resolution and contrast. The advantage of the ASC method is better reflected in the imaging of this sample. The side length of subset clearly sketches the line grating structures (Fig. 9j). As are shown in Fig. 9g–i, a clear line grating structure with 12 periods can be shown and it significantly improves the CNR. In this measurement with the condition of poor SNR, a fixed subset choice performs very poorly even if using a denoising technique. An adaptive subset choice can reconstruct the sample information without additional denoising technique.

## References

[1] Berujon S, Ziegler E, Cerbino R, Peverini L (2012). Two-dimensional X-ray beam phase sensing. Phys. Rev. Lett..

[2] Berujon S, Wang H, Pape I, Sawhney K (2013). X-ray phase microscopy using the speckle tracking technique. Appl. Phys. Lett..

[3] Morgan K, Paganin D, Siu K (2012). X-ray phase imaging with a paper analyzer. Appl. Phys. Lett..

[4] Wilkins SW, Guretev TE, Gao D, Pogany A, Stevenson AW (1996). Phase-contrast imaging using polychromatic hard X-rays. Nature.

[5] Weitkamp T, Diaz A, David C (2005). X-ray phase imaging with a grating interferometer. Opt. Express.

[6] Berujon S, Wang H, Sawhney K (2012). X-ray multimodal imaging using a random-phase object. Phys. Rev. A.

[7] Wang H, Kashyap Y, Sawhney K (2015). Hard-X-ray directional dark-field imaging using the speckle scanning technique. Phys. Rev. Lett..

[8] Wang H, Kashyap Y, Sawhney K (2016). Quantitative X-ray dark-field and phase tomography using single directional speckle scanning technique. Appl. Phys. Lett..

[9] Zdora M (2018). State of the art of X-ray speckle-based phase-contrast and dark-field imaging. J. Imaging.

[10] Kashyap Y, Wang H, Sawhney K (2016). Experimental comparison between speckle and grating-based imaging technique using synchrotron radiation X-rays. Opt. Express.

[11] Wang H, Kashyap Y, Sawhney K (2016). From synchrotron radiation to lab source: Advanced speckle-based X-ray imaging using abrasive paper. Sci. Rep..

[12] Pfeiffer F (2017). X-ray ptychography. Nat. Photon..

[13] Wang F, Wang Y, Wei G, Du G, Xue Y (2017). Speckle-tracking X-ray phase-contrast imaging for samples with obvious edge-enhancement effect. Appl. Phys. Lett..

[14] Pan B, Qian K, Xie H, Asundi AK (2009). Two-dimensional digital image correlation for in-plane displacement and strain measurement: A review. Meas. Sci. Technol..

[15] Pan B, Xie H, Wang Z, Qian K, Wang Z (2008). Study on subset size selection in digital image correlation for speckle patterns. Opt. Express.

[16] Zhou T, Zdora M-C, Zanette I, Romell J, Hertz HM (2016). Noise analysis of speckle-based X-ray phase-contrast imaging. Opt. Lett..

[17] Djurovic I, Stankovic L (2003). Adaptive windowed Fourier transform. Signal Proc..

[18] Huang L, Kemao Q, Pan B, Asundi AK (2010). Comparison of Fourier transform, windowed Fourier transform, and wavelet transform methods for phase extraction from a single fringe pattern in fringe projection profilometry. Opt. Laser. Eng..

[19] Liu J, Iskander M (2004). Adaptive cross correlation for imaging displacements in soils. J. Comput. Civil Eng..

[20] Sidick E, Green JJ, Morgan RM, Ohara CM, Redding DC (2008). An adaptive cross-correlation algorithm for extended-scene Shack–Hartmann wavefront sensing. Opt. Lett..

[21] Berujon S, Ziegler E (2015). Near-field speckle-scanning-based X-ray imaging. Phys. Rev. A.

[22] Goodman JW (2004). Introduction to Fourier Optics.

[23] Tian N, Jiang H, Li Ai, Liang D, Yan S, Zhang Z (2020). Influence of diffuser grain size on the speckle tracking technique. J. Synchrotron Radiat..

[24] Berujon S, Wang H, Alcock S, Sawhney K (2014). At-wavelength metrology of hard X-ray mirror using near field speckle. Opt. Express..

